# Temporal shifts and temperature sensitivity of avian spring migratory phenology: a phylogenetic meta‐analysis

**DOI:** 10.1111/1365-2656.12612

**Published:** 2016-12-28

**Authors:** Takuji Usui, Stuart H. M. Butchart, Albert B. Phillimore

**Affiliations:** ^1^ Institute of Evolutionary Biology University of Edinburgh King's Buildings Edinburgh EH9 3JT UK; ^2^ BirdLife International David Attenborough Building, Pembroke Street Cambridge CB2 3QZ UK; ^3^ Department of Zoology University of Cambridge Downing Street Cambridge CB2 3EJ UK

**Keywords:** arrival date, bird migration timing, climate change, migratory phenology, plasticity

## Abstract

1. There are wide reports of advances in the timing of spring migration of birds over time and in relation to rising temperatures, though phenological responses vary substantially within and among species. An understanding of the ecological, life‐history and geographic variables that predict this intra‐ and interspecific variation can guide our projections of how populations and species are likely to respond to future climate change.
2. Here, we conduct phylogenetic meta‐analyses addressing slope estimates of the timing of avian spring migration regressed on (i) year and (ii) temperature, representing a total of 413 species across five continents. We take into account slope estimation error and examine phylogenetic, ecological and geographic predictors of intra‐ and interspecific variation.
3. We confirm earlier findings that on average birds have significantly advanced their spring migration time by 2·1 days per decade and 1·2 days °C^−1^. We find that over time and in response to warmer spring conditions, short‐distance migrants have advanced spring migratory phenology by more than long‐distance migrants. We also find that larger bodied species show greater advance over time compared to smaller bodied species. Our results did not reveal any evidence that interspecific variation in migration response is predictable on the basis of species' habitat or diet.
4. We detected a substantial phylogenetic signal in migration time in response to both year and temperature, suggesting that some of the shifts in migratory phenological response to climate are predictable on the basis of phylogeny. However, we estimate high levels of species and spatial variance relative to phylogenetic variance, which is consistent with plasticity in response to climate evolving fairly rapidly and being more influenced by adaptation to current local climate than by common descent.
5. On average, avian spring migration times have advanced over time and as spring has become warmer. While we are able to identify predictors that explain some of the true among‐species variation in response, substantial intra‐ and interspecific variation in migratory response remains to be explained.

## Graphical abstract

Advances in avian migration phenology over time and with rising temperatures exhibit substantial intra‐ and interspecific variation. The authors conduct an extensive phylogenetic meta‐analysis allowing us to identify key predictors and variance components of the phenological response, and whether this sensitivity to temperature increase may be phylogenetically conserved.

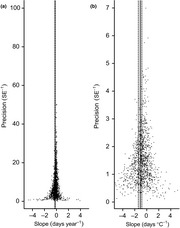

## Introduction

Changes in the timing of seasonal events are one of the most conspicuous biotic impacts of global climate change (Walther *et al*. 2002; Parmesan & Yohe 2003; Root *et al*. 2003). Rising global temperatures have generally resulted in an earlier onset of spring in extra‐tropical regions (Thackeray *et al*. 2016), including earlier flowering and leafing of plants (Fitter & Fitter 2002), emergence of insects (Roy & Sparks 2000), and breeding of amphibians and birds (Crick *et al*. 1997; Li, Cohen & Rohr 2013). As a result of high levels of interest from professionals and citizen scientist ornithologists, temporal shifts in the timing of spring avian migration due to climate change have been especially extensively recorded over time and space (BirdLife International & National Audubon Society 2015). These studies reveal a general trend of advancement in spring arrival and passages dates towards the present and with increasing temperatures (Lehikoinen, Sparks & Zalakevicius 2004; Lehikoinen *et al*. 2010). However, there is considerable variation in the slope around the average, with different species or even populations of the same species exhibiting both earlier and later timings of spring migration over time and with respect to temperature change (Miller‐Rushing, Primack & Stymeist 2008; Hurlbert & Liang 2012). Such variation in phenological response has potentially severe consequences on mean population fitness if migratory birds arrive at breeding grounds too early or late relative to peak resource availability, resulting in mismatch of trophic interactions (Both *et al*. 2009; Thackeray *et al*. 2010) or stronger competition for finding optimal breeding sites or high‐quality mates (Alatalo, Lundberg & Glynn 1986; Smith & Moore 2005). Indeed, there is some evidence that migrant bird species are declining by more than residents (Both *et al*. 2006; Møller, Rubolini & Lehikoinen 2008), with mistiming of breeding among the potential explanations.

What factors might cause populations and species to vary in their phenological response? Comparative analyses can be used to reveal the factors that explain trait variation among (Harvey & Pagel 1991) or within (Stone, Nee & Felsenstein 2011) species. Previous comparative studies addressing avian migratory responses have found that short‐distance migrants are more responsive to spring temperatures than long‐distance migrants (e.g. Butler 2003; Lehikoinen, Sparks & Zalakevicius 2004; Rubolini *et al*. 2007). This pattern may be explained if conditions in the non‐breeding and passage ranges of short‐distance migrants are more predictive of conditions on the breeding grounds than is the case for long‐distance migrants. In contrast, long‐distance migrants overwintering further away from the breeding grounds may rely on circannual, endogenous mechanisms to time their migration rather than external cues (Gwinner 1996). However, although a difference in response according to migration distance has been widely reported, the pattern has been far from universal (Jonzén *et al*. 2006; Zalakevicius *et al*. 2006).

Other ecological traits that have recently been suggested to predict phenological response to climate change include species' habitat and diet type. In particular, the timing of leaf out in temperate forests has advanced substantially in response to rising spring temperatures, which in turn impacts on the timing of the peak availability of some herbivorous invertebrates (Visser & Both 2005). Forest habitats may be more highly seasonal in phytophageous invertebrate availability than other habitats such as marsh/reeds: In the former, invertebrate availability is restricted to before the production of secondary plant compounds (Feeny 1970; Southwood *et al*. 2004), whereas in the latter, reed continues to grow during the spring and summer, and hence invertebrate availability may be less seasonally peaked (Halupka, Dyrcz & Boroweic 2008). It follows therefore that migrants that feed these highly seasonal resources to offspring may experience strong selection to track environmental changes (Visser *et al*. 1998; Both *et al*. 2010). Similarly, if species that are specialists in terms of diet, habitat or climatic niche experience stronger selection on migration timing then they may have steeper phenological responses (Both *et al*. 2010). Running counter to this prediction, however, previous comparative studies have found that generalist species are more responsive than specialists to climate change (Végvári *et al*. 2010; Moussus *et al*. 2011; Hurlbert & Liang 2012). Additionally, body size is expected to be negatively correlated with the magnitude of advance in spring migratory phenology, as migration in larger birds is hypothesized to be more time‐canalized due to longer moulting times and slower migration speeds (Hedenström 2006, 2008).

The timing of migration is expected to affect fitness, and a change in phenology over time may be due to either a response to selection or phenotypic plasticity. However, few studies present compelling evidence for microevolution of phenology in response to recent climate change [see Franks, Sim & Weis (2007) for a plant flowering time example], and most recent changes in migratory phenology are thought to be attributable to phenotypic plasticity (reviewed in Charmantier & Gienapp 2014). However, it is quite possible that microevolution in the form of local adaptation contributes to among‐species and population variation in the plastic response to spring temperatures. We predict that, even in the absence of among‐site or among‐species variation in the plastic response to temperature, geographic variation in the rate of temperature increase will generate geographical variation in the average phenological response. Consistent with this prediction, there is evidence that the magnitude of change in migratory phenology varies latitudinally and is steepest for high latitude areas that have experienced the greatest temperature increases (Sparks & Braslavská 2001; Parmesan 2006; but see Rubolini *et al*. 2007). More generally, studies conducted in the northern hemisphere have reported differences in slope of migratory response between continents, reflecting regional differences in climatic change (Bitterlin & Van Buskirk 2014).

Previous comparative studies addressing avian migratory phenology have been restricted to the northern hemisphere (e.g. Rubolini *et al*. 2007; Végvári *et al*. 2010; Bitterlin & Van Buskirk 2014), although there has been a recent increase in studies of southern hemisphere species (reviewed in Chambers, Beaumont & Hudson 2014). Beyond simply correcting for phylogenetic non‐independence (Felsenstein 1985), most phylogenetic comparative studies now estimate phylogenetic signal, i.e., the extent to which close relatives share similar traits (Freckleton, Harvey & Pagel 2002; Blomberg, Garland & Ives 2003). This can be useful as a predictive tool, as a strong phylogenetic signal implies that we might predict the phenological responses of species not included in the study based on the responses of their close relatives. Of the avian studies that have incorporated phylogeny, two report the phylogenetic signal to be low (Végvári *et al*. 2010; Bitterlin & Van Buskirk 2014), while one reports the signal to be high (Rubolini *et al*. 2007). However, these studies ignore measurement error in slope estimates, meaning that the residual variance in the slopes is likely to be inflated and the phylogenetic signal underestimated.

Here we conduct a phylogenetic meta‐analysis of avian migratory phenology on a global‐scale, with the aim of identifying key predictors of global variation in slope of (i) the temporal phenological trend and (ii) the phenological response to temperature, while controlling for aspects of study methodology. Specifically, on the basis of previous evidence and theory outlined above, we hypothesize steeper slopes for migrants that (i) are short‐distance migrants, (ii) rely on forest breeding and passage habitats and (iii) have an invertebrate‐dominated diet. We also hypothesize that (iv) greater species' generalism in terms of habitat and diet will give rise to shallower slopes, (v) body size will correlate negatively with phenological trends, such that the smallest species advance by most, and (vi) the magnitude of the slopes will increase with latitude. Finally, we hypothesize that (vii) phenological responses will be phylogenetically conserved.

## Materials and methods

### Data selection and criteria

We conducted a systematic literature search in order to locate relevant studies, by following the PRISMA (Preferred Reporting Items for Systematic reviews and Meta‐analyses) statement (http://www.prisma-statement.org). We searched for studies by using key words ‘avian’ or ‘bird’ with ‘migration phenology’, ‘arrival date’ or ‘timing of migration’ on *ISI Web of Science* and *Scopus*. To each combination of the search string, we added regional terms including ‘Southern hemisphere’, ‘Africa’, ‘Asia’, ‘South America’, ‘Antarctic’ or ‘Australia’, with the aim of increasing the representation of species from these regions given a predominance of migration studies from North American and European localities. Additionally, searches were carried out on *Google Scholar* to locate missing publications and ‘grey‐literature’ (e.g. unpublished material and dissertations). All searches were carried out between January and October 2015.

We extracted data from studies reporting changes in spring migratory phenology over time or with respect to temperature. Specifically, we extracted data from studies reporting either the slope and standard error from a simple linear regression of a measure of spring migration timing against year (days year^−1^) or temperature (days °C^−1^). Where studies did not report the standard error but reported the slope, sample size and *P*‐value of the linear regression analysis, we calculated an upper estimate of the standard error by (i) calculating the *t*‐value (where *P* was reported as <0·05 or <0·01 we used 0·05 or 0·01 respectively) and (ii) dividing the slope by the *t*‐value. Where studies did not report the above, we contacted authors for data sets, from which slopes and standard errors were extracted using linear regression. In a few cases, upon contact, authors supplied us with additional data sets. For papers where the requisite data were presented in graphical format, we extracted data points using WebPlotDigitizer v3.9 (Rohatgi 2015; http://arohatgi.info/WebPlotDigitizer) and re‐analysed the data using linear regression. Finally, we included reports that presented annual bird arrival dates to the breeding or passage grounds but did not estimate a trend over time, by calculating this using linear regression.

We included studies that reported changes in first arrival dates (FADs) or mean/median arrival dates (MADs) to the breeding or passage grounds. For temperature response slopes, we included changes in spring migratory phenology with respect to temperature changes at breeding, passage or non‐breeding sites. Where a study had considered temperature across multiple time periods for a population, we included only the highest *R* ^2^ correlate for each temperature location. All studies used in the meta‐analysis are provided in the Data Sources section. The full data set can be accessed from the Dryad Digital Repository: http://dx.doi.org/10.5061/dryad.mb4nd. In general, the slope estimates we obtained from the literature assume that the migration time within a year is known without error. We anticipate that ignoring such error will not bias slope estimates based on mean/median but will lead to underestimation of the standard error associated with a slope. However, ignoring this source of uncertainty may bias slopes estimated from first dates if either abundance or recorder effort has changed over time and/or with temperature.

### Location and species traits

We collected data on geographic and species' ecological and life‐history traits that have been suggested to influence the strength of phenological response of birds to climate change (see Introduction). For each study, we defined geographic factors by the latitude, longitude, country and continent of the study site. In cases where data were collected on a regional scale, we calculated the mid‐point coordinates for the study site.

At the population‐level, we classified bird migration distances as either short‐ or long‐distance migrants, on the basis of statements made by authors of the studies. Due to intraspecific differences in species' migration distances, we only assigned migration distances to migrants when this was reported in papers for the population under study. Where studies referred to a population as medium‐distance migrants, these were reclassified as short‐distance migrants if their breeding and non‐breeding grounds were within the same continent. Species‐level data on habitat was obtained from BirdLife International (2015). We constructed a binary classification of species as having ‘forest’ or ‘other’ habitat, depending on whether species used forest habitats as a suitable breeding or passage habitat or not. Habitat generalism was quantified as the number of different suitable breeding and passage habitats (at the highest level in the IUCN Habitats classification scheme; http://www.iucnredlist.org/technical-documents/classification-schemes/habitats-classification-scheme-ver3) used by a species. Habitat types comprised forest, shrubland, grassland, wetland, marine, savanna, desert, rock, cave, and artificial aquatic and terrestrial habitats. Data on species' diet were obtained from Wilman *et al*. (2014), with diet categories comprising invertebrates, fish, reptiles and amphibians, mammals and birds, general or unknown vertebrates, fruits, seeds, nectar and pollen, other plant materials or carrion. We classified species as ‘invertebrate‐dominated’ where invertebrates comprised a majority (≥50%) of the diet, with the remainder classified as ‘other’. Additionally, we scored diet generalism for each species by counting the number of food types that built up a substantial (≥20%) component of a species' diet. We also obtained data on the mean body mass (g) for each species, as reported in Dunning (1992) and Wilman *et al*. (2014).

### Statistical analysis

We examined signs of publication bias (the preferential publication of statistically significant results) in the data set indirectly by visualization of funnel plots. If there is no bias, plotting slope estimates against a measure of precision (inverse of the standard error) should show a symmetrical and inverted funnel, with smaller studies showing larger variance (Egger *et al*. 1997). Where present, publication bias can generate unreliable meta‐analytical results (Sterne & Egger 2001).

For analysis of slopes and standard errors, we adopted a mixed effects phylogenetic meta‐analytic approach, in which effects of multiple fixed and random effects can be specified in a single model (Hadfield & Nakagawa 2010; Nakagawa & Santos 2012). Analyses were implemented in a Bayesian setting using the package MCMCglmm (Hadfield 2010) in R (R Development Core Team 2014). We sampled 100 sample trees from a pseudo‐posterior distribution of species‐level bird phylogenies (Jetz *et al*. 2012) from BirdTree.org (http://www.birdtree.org). Trees were based on the Hackett *et al*. (2008) backbone. We fitted phylogeny, species, study and location as random effects (eqn 1).

(eqn 1) $$y_{i}=\mu +\beta x_{i}+a_{i}+s_{i}+t_{i}+l_{i}+s_{i}:l_{i}+e_{i}+m_{i}$$

The Gaussian trait, *y* (estimate of the slope of phenology regressed on year or temperature), of species *i* is given by the grand mean (μ) plus the influence of fixed effects (β*x*_*i*_), and random effects due to phylogeny (*a*_*i*_), species (*s*_*i*_), study (*t*_*i*_), location (*l*_*i*_), each species by location combination (*s*_*i*_ : *l*_*i*_), residual (*e*_*i*_) and measurement error (*m*_*i*_). All random effects were assumed to follow normal distributions and their variances were estimated (with the exception for *m*_*i*_ for which variance was fixed at 1). As arrival data from studies included in this meta‐analysis originated from various sources (citizen scientist observations; ornithological club reports; observatory observations; standardized capture and ringing at observatories; non‐standardized field studies; and standardized field studies), we allowed for heterogeneity in residual variance across these data types to control for variance in the quality of data in all our models. Therefore, the residual variance for each data type reveals how much the slope estimates depart from the average slope obtained for population *i*. We calculated the per cent variance for each random effect component by dividing estimates of each variance component by total variance (calculated as the sum of phylogeny, species, study, location, species by location and the mean of the residual variance terms).

Fitting phylogeny as a random effect accounts for non‐independence among species due to shared history under the Brownian motion model of trait evolution. We assume that different metrics (e.g., FAD and MAD) share the same phylogenetic signal. Measurement error variance, defined as the squared standard error of the slope estimate for migration regressed on year or temperature, ensured that more reliable estimates were given more weight in the model. By repeating the analyses across 100 trees, the combined posterior distribution for fixed and random effects capture both model and phylogenetic uncertainty (Pagel & Lutzoni 2002). We estimated the phylogenetic heritability (*H* ^2^), which is mathematically equivalent to calculating pedigree‐based heritability in quantitative genetics (Hadfield & Nakagawa 2010), as:

(eqn 2) $$H^{2}=\sigma_{a}^{2}/\left(\sigma_{a}^{2}+\sigma_{s}^{2}\right)$$

where $\sigma_{a}^{2}$ is phylogenetic variance and $\sigma_{s}^{2}$ is species variance. For each type of slope estimate (year and temperature), we constructed three main types of models: (i) a null model – with the objective of estimating the global mean advance in migration timing, (ii) a basic model – with the objective of estimating sources (e.g. spatial, species, phylogenetic) of variance in true migration slopes around the global mean while controlling for aspects of study methodology and (iii) an ecological model – with the objective of identifying ecological predictors of migration trends and temperature sensitivity.

In addition to the above random effects (eqn 1), the basic models included the following fixed effects to control for aspects of study methodology: the metric for monitoring spring migration timing (FAD or MAD), location of migrants' arrival (breeding or passage ground), location of temperature data (breeding, passage or non‐breeding ground) and the midyear decade in which the study was conducted. Midyear decade was included in the year response models to account for imbalance in data coming from different periods and to control for any tendency in the slope to steepen in decades that experienced greater directional temperature change. The ecological models included, in addition to the fixed terms included in the basic model, the continent of the study site; an interaction between hemisphere and latitude of the study site; migration distance category; invertebrate diet binary score; diet generalism; forest habitat binary score; habitat generalism; and ln body mass. Preliminary analysis revealed a strong correlation between location of migrants' arrival and location of temperature data, and between continental and latitudinal effects, and thus we considered these predictors in separate models. We also included the source of data as a fixed effect, to allow for a bias in slope estimates for first dates if recorder effort has changed over time.

MCMCglmm models were run for 200 000 iterations on each tree, discarding the first 150 000 iterations as burn‐in, and sampling every 500 iterations. For random effects, an inverse Wishart prior with *V *=* *1 and *nu* = 0·02 was specified (Gelman & Hill 2007). We viewed trace plots for fixed and random effects to ensure appropriate sampling of the posterior distribution and ensured that effective sample sizes for all parameters exceeded 1000. We deemed fixed effects to be statistically significant when 95% credible intervals (CIs) did not span zero.

## Results

### Data set coverage and bias

**Figure 1:**
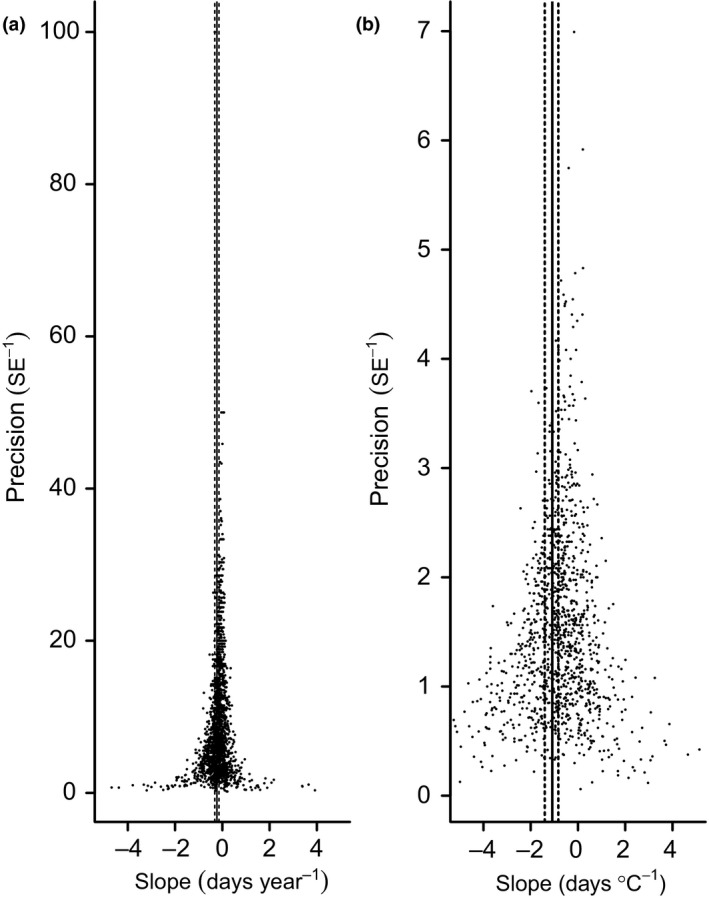
Funnel plots of slope estimates plotted against the inverse of standard errors (SE) obtained (a) over time (days year^−1^) and (b) with respect to temperature (day °C^−1^). Vertical lines represent the average effect size (solid) for the slope of spring migration timing and the associated lower and upper 95% CIs (dashed), as estimated using a mixed model meta‐analysis that included phylogeny, species, study and location as random effects and the grand mean as the sole fixed effect.

Our final data set consisted of 2976 slope estimates, comprising 1816 year slopes and 1160 temperature slopes obtained from 73 published studies. Observations spanned a period of 265 years from 1749 to 2014, with most studies focusing on migration trends in the past 50 years. Observation duration ranged from 5 to 72 years, with a mean duration of 38·1 years. The data set included 413 species representing 28 orders, with Passerines comprising 73·9% of the total data set (Table S1, Supporting Information). Substantial geographic bias was evident, with data from North America and Europe accounting for 52·8% and 28·3% of the overall data set, while the southern hemisphere accounted for just 3·9% (Table S2). Notably, none of the southern hemisphere studies reported temperature response slopes. Visual examination of funnel plots suggested no evidence of publication bias in either the year or the temperature slopes (Fig. 1).

### Correlates of year response

**Figure 2:**
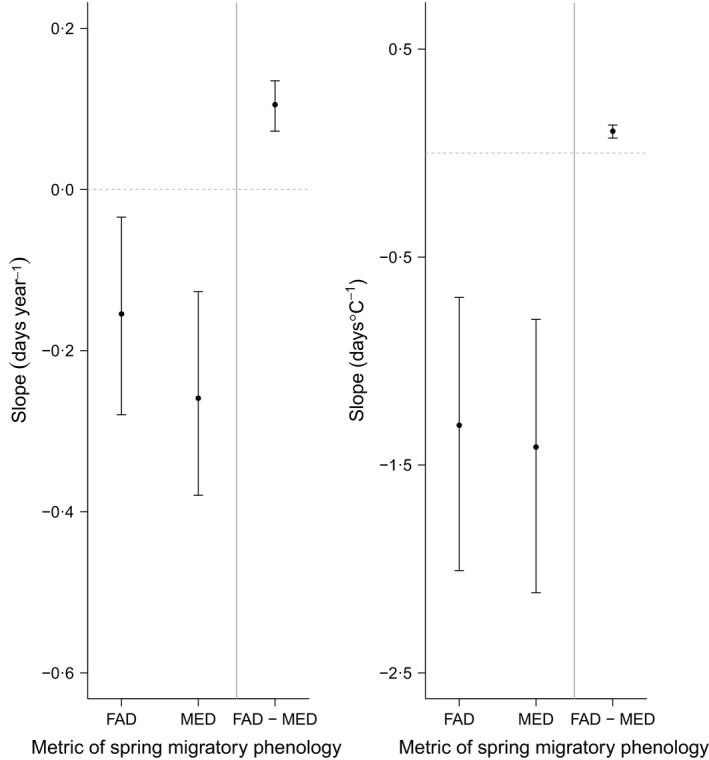
Posterior median advances (and associated 95% CIs) in spring migration timing (a) over time (days year^−1^) and (b) with respect to temperature (day °C^−1^) for different metrics of monitoring migration timing, as estimated under the basic model. Estimates are for arrival to the breeding ground as reported by standardized field studies, with year response estimates representing advances in the decade 1980. Note that although CIs of the FAD and MAD slope estimates overlap each other, the 95% CI for the difference in slope between FADs and MADs as estimated directly from the basic models (plotted to the right of the grey vertical line) does not overlap zero and is significant.

We found a highly significant trend for earlier spring migration timing over time (Fig. 1a). Overall, the global average advance in migration timing estimated by the null model was −2·1 (95% CI: −2·9 to −1·4) days per decade. In all models, this advance in migration timing was significantly steeper in MADs than for FADs (Fig. 2a; Table S3). Furthermore, in all models, the advance in migration timing varied among decades, being steepest in the 1920s and 1990s (Table S3). Advance in migration timing did not differ significantly between arrival at breeding or passage grounds, or between different data types.

The basic model revealed a significant phylogenetic signal, *H* ^2^ = 0·672 (95% CI: 0·321 to 0·942). Phylogeny and species contributed around 11·4% and 5·6% (i.e. 17·0% among‐species variance) of total variance in the slope, respectively, with the lower CI close to zero for both components. Intraspecific variance, captured by location and species by location variance accounted only for around 5·3% and 3·8% (i.e. 9·1% within‐species variance) of total variance, respectively, with again the lower CI close to zero for both components (Table S3). Among‐study and mean residual variance accounted for around 27·6% and 46·4% of total variance, respectively, and were significant sources of variation (Table S3). The magnitudes of variance components were qualitatively similar for models that included ecological and life‐history correlates as fixed effects.

**Figure 3:**
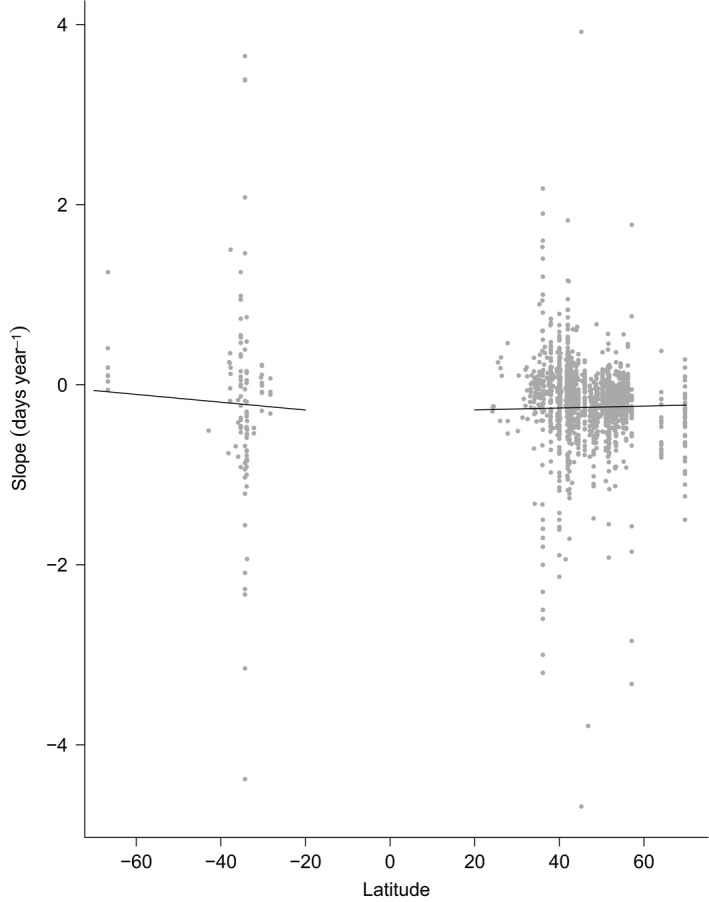
Predicted effects of latitude on changes in mean/median arrival dates over time in the Northern and Southern Hemispheres. Grey circles represent temporal slope estimates. Black lines represent the latitudinal predictions in the Northern and Southern Hemispheres, as estimated under the ecological model with latitude as a predictor. Estimates are for short‐distance migrants; migrants that do not rely on forest habitats during breeding and passage; migrants with a predominantly invertebrate diet; habitat and diet specialists; body size of 10 g; arrival data as reported by standardized field studies; and the decade 1980.

**Figure 4:**
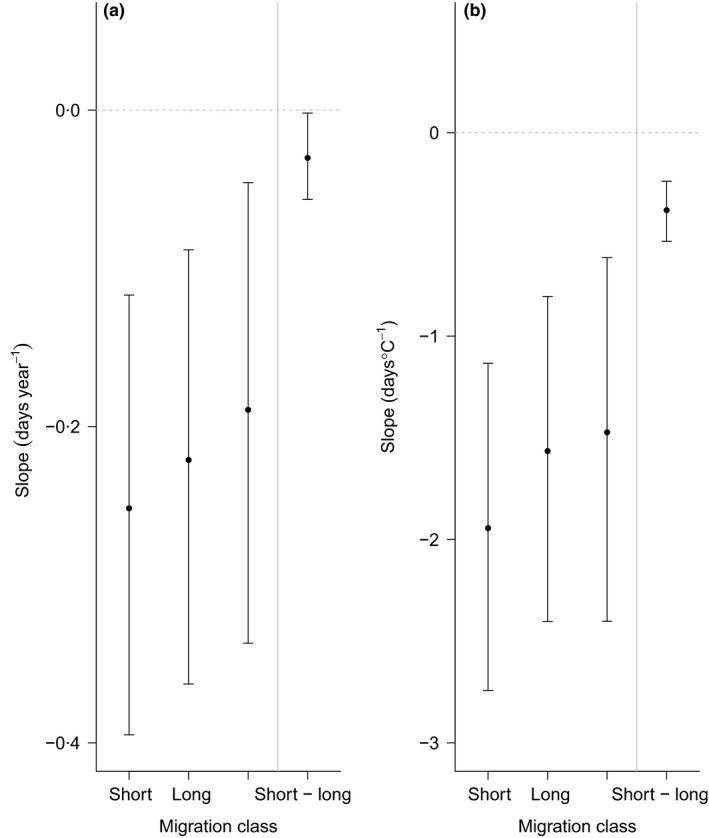
Posterior median advances (and associated 95% CIs) in mean/median arrival dates for different migration distance classes (a) over time (day year^−1^) and (b) with temperature (days °C^−1^) at the mean latitude of the data set in the Northern Hemisphere (46·1°N), as estimated under our ecological models. Unclassified migrants refer to migrants that were not assigned migration distances in the original studies. Estimates are for migrants that do not rely on forest habitats during breeding and passage; migrants with a predominantly invertebrate diet; habitat and diet specialists; body size of 10 g; arrival data as reported by standardized field studies; and the decade 1980 for year slope estimates. The difference in slope between short‐ and long‐distance migrants as estimated directly from our ecological models (plotted to the right of the grey vertical line) does not overlap zero and is significant.

Under the ecological models, we found no significant latitudinal trends in year slopes (Fig. 3) or differences among continents. We found that short‐distance migrants have advanced their migration timing by significantly more than long‐distance migrants, although long‐distance migrants still showed a significant advance in most decades (Fig. 4a). Additionally, the magnitude of the negative slope was found to increase very slightly with species' body size. From the ecological model including latitude, we estimate that with an increase in body mass from 10 to 1000 g, the slope steepens by 0·04 days year^−1^. We found no significant effect of habitat, diet or either index of species' generalism (Table S3).

### Correlates of temperature response

We found a highly significant trend for earlier spring migration at higher temperatures under the null model (Fig. 1b). Overall, the posterior median advance in migration timing was −1·2 (95% CI: −1·7 to −0·9) days °C^−1^. While this appears substantially more negative than the mean of the funnel plot, this can be attributed to random effects relating to region and study. In all models, the advance in migration timing was significantly steeper in MADs compared to FADs (Fig. 2b). The slope of response did not vary depending on whether temperatures were representative of changes at breeding, passage or non‐breeding sites, whether arrival was to the breeding or passage grounds, or across different data types (Table S4).

We detected a significant phylogenetic signal in temperature slopes (*H* ^2^ = 0·468 [95% CI: 0·140 to 0·984]). In our basic model, phylogeny and species accounted for around 13·0% and 14·6% (i.e. 27·6% among‐species variance, as estimated from our basic model; Table S4) of total variance in the slope of phenology on temperature, respectively, with lower CI close to zero for species variance. Intraspecific variance, captured by location and species by location components accounted for 29·6% and 1·7% (i.e. 31·3% within‐species variance) of total variance in the slope, respectively, with lower CI for both components close to zero. Among‐study and mean residual variance accounted for around 19·2% and 21·9% of total variance, respectively, and were significant sources of variation (Table S4). The magnitudes of variance components were qualitatively similar across our ecological models with the exception of location variance, which decreased when continent of the study location was included as a correlate, and among‐study variance, which, as a percentage of total variance in the slope, decreased in all ecological models (Table S4).

Under the ecological model, we found no significant latitudinal trends in the temperature slope (Table S4). As with the year response meta‐analysis, short‐distance migrants showed significantly more negative responses as compared with long‐distance migrants, although long‐distance migrants still showed a significant response overall (Fig. 4b). We found no significant effect of body size, habitat, diet or either index of species' generalism (Table S3).

## Discussion

We present the most phylogenetically and geographically extensive meta‐analysis to date on changes in avian spring migratory phenology. Our results agree with previous findings that in recent decades, the average avian spring migration timing has advanced in response to climate change (Parmesan & Yohe 2003; Root *et al*. 2003; Lehikoinen, Sparks & Zalakevicius 2004; Lehikoinen *et al*. 2010), with migrants arriving at their breeding grounds on average 2 days per decade earlier. This estimate of migration phenology is in broad agreement with estimates derived from previous meta‐analyses with partially overlapping data sets (Lehikoinen, Sparks & Zalakevicius 2004; Gienapp, Leimu & Merilä 2007; Rubolini *et al*. 2007; Bitterlin & Van Buskirk 2014). Our results also confirm that migration timing is temperature sensitive, with migrants arriving around 1 day earlier 1 °C^−1^ rise in global temperatures. Importantly, however, our results reveal substantial heterogeneity in the true magnitude and sign of phenological response to climate change across phylogeny, species and populations.

Our results highlight species' migration distance as a key correlate of variation in strength of phenological response. Our finding of steeper temporal and temperature slopes for short‐ compared with long‐distance migrants confirms previous findings that the former may be better able to evolve plastic responses that partially track changes in climatic conditions at their breeding grounds (e.g. Butler 2003; Lehikoinen, Sparks & Zalakevicius 2004). We note that long‐distance migrants are also able to significantly adjust their migratory phenology in response to climate change, suggesting that climatic variables used as cues to time migration at their non‐breeding grounds could covary with climatic conditions at the breeding grounds (Gordo *et al*. 2005; Saino & Ambrosini 2008) and/or that migrants are able to adjust their migration speed in response to warmer conditions during passage (Marra *et al*. 2005). Life‐history traits that are correlated with body size, such as moulting and migratory speed, have been shown to affect phenological response to climate change including in the most recent meta‐analysis by Bitterlin & Van Buskirk (2014). We also find that body size has a negative relationship with migration time, such that the largest bodied species are advancing most strongly, although this finding is contrary to our expectation that larger birds may be more time constrained in their response to climate change (Hedenström 2006, 2008).

We do not detect differences in response between diet type or habitat generalists and specialists. Although studies conducted in both North America and Europe have revealed stronger responses for species that are more generalist in terms of diet and climatic niche (Végvári *et al*. 2010; Hurlbert & Liang 2012), our results suggest that generalism is at best a weak correlate of changes in migration phenology. We also do not find a significantly steeper slope for forest inhabiting species or for species with an invertebrate‐dominated diet. We do not rule out the possibility that habitat and diet may still have an effect on species' migration timing, however, and note that other habitat or food types may show similarly peaked seasonal availability. For example, in a study of American migrants, Butler (2003) found that grassland species advanced their spring migration timing the most, perhaps due to earlier snow melt allowing earlier availability of seeds for which most of these migrants rely on.

Although spatial variance in both responses was estimated to be quite large, we find no latitudinal trend in migration slopes, counter to claims of steeper temporal slopes (Sparks & Braslavská 2001; Parmesan 2006) and greater temperature responsiveness with latitude (Both *et al*. 2004; While & Uller 2014) in the northern hemisphere. However, previous findings from a global meta‐analysis of various plant and animal species have estimated latitude to account for less than 4% of variation in response (Root *et al*. 2003; Parmesan 2007). As a percentage of total variance, among‐location variance was lower for the year response than the temperature response. We suggest that among‐location variance and among‐study variance may capture similar effects: The summed proportion of location and study variance accounts for 36·7% and 50·5% of total variance in year and temperature slopes respectively. This combined spatial variance component corresponds to 95% of true advances in migration timing varying between −14·0 days per decade and 9·8 days per decade over time and −15·1 days °C^−1^ and 12·7 days °C^−1^ with temperature. We find this summed variance to be a significant source of variation in all our year and temperature slope analyses, suggesting that considerable geographical heterogeneity exists for both temporal shifts and temperature sensitivity in migratory phenology. Some of this variation may be attributable to a plastic response to geographical variation in environmental or biotic drivers that we have overlooked (Gordo *et al*. 2005; Saino & Ambrosini 2008), or due to genetic differences among populations if they are locally adapted in their plastic response to temperature (see also While & Uller 2014).

Our finding of substantial phylogenetic signal in the year and temperature slopes is in contrast to a recent informal meta‐analysis on changes in migratory phenology in the northern hemisphere (Bitterlin & Van Buskirk 2014). Our estimate of significant phylogenetic signal reveals that there may be some phylogenetic constraints to the timing of migration in response to climate, consistent with findings in plants (Willis *et al*. 2008) and butterflies (Roy *et al*. 2015). However, high levels of species and spatial variance estimates for the temperature response model are consistent with a scenario where plasticity in response to climate evolves rapidly and is more influenced by adaptation to current local climate than by common descent (Rubolini *et al*. 2007; Végvári *et al*. 2010). For this reason, we suggest that phylogenetic relationships are likely to be of little value in predicting the migratory responses of populations of further species.

Finally, as a variety of approaches exist for estimating migration time response, it is desirable to control for these differences and distinguish between true variation in phenological response and artefacts caused by differences in field methodology. Estimates of residual variance components in both year and temperature slopes are large, which suggests considerable heterogeneity in slope estimates arises due to different data collection methods of migrants' arrival dates. We also find differences in response between different metrics of spring migration timing, consistent with the explanation that FADs are subject to biases such as changes in sampling effort and population size, making them less reliable than measures of average arrival dates for estimating population phenological trends (Sparks, Roberts & Crick 2001; Tryjanowski & Sparks 2001; Rubolini, Saino & Møller 2010). We suggest that steeper slopes for changes in MADs compared with FADs may result from a decline in migrant population sizes (Robbins *et al*. 1989; Sanderson *et al*. 2006) causing first dates to be delayed relative to MADs. Additionally, we did not find significant differences in migratory phenology to temperature changes at breeding compared to non‐breeding grounds. Unfortunately, studies that consider climatic data from non‐breeding grounds (e.g. from Central and South America) remain scarce relative to those of breeding grounds (comprising just 10·6% of our temperature records).

Our estimate of an average migratory response to temperature of −1·2 days °C^−1^ is shallower than estimates of the response of lay dates to spring temperatures (Dunn & Møller 2014; Phillimore *et al*. 2016; Thackeray *et al*. 2016). If spring temperatures continue to rise, this shallow response of migration time to spring temperature may act as a hard limit on the lay date response to breeding ground temperatures, thus placing migrant species at a disadvantage relative to resident species (Both & Visser 2001). Such demographic consequences have been reported for a Dutch population of pied flycatchers, where population declines have been most severe in areas with an early food peak and for migrants that are least flexible in their response to temperature increases (Both & Visser 2001; Both *et al*. 2006). In addition, our finding that long‐distance migrants are less responsive to rising temperatures than short‐distance migrants present grounds for concern. If spring temperatures on the breeding ground continue to rise, advances in the timings of plant and invertebrate phenology and optimum lay date are also predicted (Gienapp *et al*. 2013; Vedder, Bouwhuis & Sheldon 2013).

In this phylogenetic meta‐analysis, we find that the average migratory bird is returning to breeding grounds significantly earlier than in the past and as temperatures rise. Around this mean, we identify substantial variation in the true response of different species and populations. While we identify some ecological predictors of this variation, substantial intra‐ and interspecific variation in migratory response remains to be explained. We note that although our data set is extensive, it is by no means exhaustive, with our meta‐analyses predominantly comprising English‐language literature. By adopting PRISMA procedures, however, we have crucially followed a replicable process in determining which studies are to be included in the meta‐analysis, the importance of which has been stressed in ecological and evolutionary meta‐analyses (Nakagawa & Poulin 2012). We further note that inclusion of spatial random effects in our models as well as use of geographical terms in our search string mitigates spatial bias in our data set. Thus, we have presented here, to our knowledge, the most extensive, formal meta‐analyses of avian migration phenology to date.

## Data accessibility

Data available from the Dryad Digital Repository: http://dx.doi.org/10.5061/dryad.mb4nd (Usui, Butchart & Phillimore 2016).

## Supporting information


**Table S1.** Taxonomic coverage of the data set used in meta‐analysis.
**Table S2.** Geographical coverage of the data set used in meta‐analysis.
**Table S3.** Model coefficients from the analyses of year slopes (days year^−1^) in basic and ecological models.
**Table S4.** Model coefficients from the analyses of temperature slopes (day °C^−1^) in basic and ecological models.
