# Supplementary material for: Temporal shifts and temperature sensitivity of avian spring migratory phenology: a phylogenetic meta‐analysis
Source: J Anim Ecol. 2016 Dec 28;86(2):250–61. doi: 10.1111/1365-2656.12612 (PMC6849580; doi:10.1111/1365-2656.12612)
Supplement: Supplementary file 1 — Table S1. Taxonomic coverage of the data set used in meta‐analysis. Table S2. Geographical coverage of the data set used in meta‐analysis. Table S3. Model coefficients from the analyses of year slopes (days year−1) in basic and ecological models. Table S4. Model coefficients from the analyses of temperature slopes (day °C−1) in basic and ecological models. [file JANE-86-250-s001.docx]

**Supplementary Information**

1. Additional Results

2. Bayesian models

3. Data file (uploaded separately in Dryad repository)

1. Additional Results

Table S1. Taxonomic coverage of dataset used in meta–analyses. For each bird order, the number of estimates obtained for year and temperature slopes (n) are given, where
‘–’ indicates no slope estimates obtained.

| Order | Year response (n) | Temperature response (n) | Total (n) | Total (%) |
| --- | --- | --- | --- | --- |
| Accipitriformes | 20 | 13 | 33 | 1.11 |
| Alaudidae | 0 | 2 | 2 | 0.07 |
| Anseriformes | 53 | 44 | 97 | 3.26 |
| Apodiformes | 64 | 46 | 110 | 3.70 |
| Bucerotiformes | 1 | 2 | 3 | 0.10 |
| Caprimulgiformes | 7 | 3 | 10 | 0.34 |
| Cathartiformes | 3 | 2 | 5 | 0.17 |
| Charadriiformes | 123 | 82 | 205 | 6.89 |
| Ciconiiformes | 3 | 3 | 6 | 0.20 |
| Columbiformes | 26 | 10 | 36 | 1.21 |
| Coraciiformes | 18 | 1 | 19 | 0.64 |
| Cuculiformes | 85 | 47 | 133 | 4.47 |
| Falconiformes | 8 | 3 | 11 | 0.37 |
| Galliformes | 1 | 2 | 3 | 0.10 |
| Gaviiformes | 3 | 3 | 6 | 0.20 |
| Gruiformes | 13 | 11 | 24 | 0.81 |
| Motacillidae | 0 | 2 | 2 | 0.07 |
| Muscicapidae | 0 | 2 | 2 | 0.07 |
| Passeriformes | 1342 | 858 | 2200 | 73.92 |
| Pelecaniformes | 15 | 12 | 27 | 0.91 |
| Piciformes | 2 | 1 | 3 | 0.10 |
| Podicipediformes | 7 | 6 | 13 | 0.44 |
| Procellariiformes | 6 | 1 | 7 | 0.24 |
| Psittaciformes | 7 | 0 | 7 | 0.24 |
| Sphenisciformes | 1 | 0 | 1 | 0.03 |
| Strigiformes | 2 | 1 | 3 | 0.10 |
| Suliformes | 4 | 3 | 7 | 0.24 |
| Turniciformes | 2 | 0 | 2 | 0.07 |

Table S2. Geographical coverage of dataset used in meta–analyses. For each country, the number of estimates obtained for year and temperature slopes (n) are given, where
‘–’ indicates no slope estimates obtained. Russia was classified as both European and Asian depending on the study sites’ coordinates, except for those that were carried out close to the European–Asian border where upon continental location was not classified.

| Country | Year  response (n) | Temperature response (n) | Total (n) | Total (%) |
| --- | --- | --- | --- | --- |
| *Antarctica* |  |  |  |  |
| Antarctic | 8 | – | 8 | 0.27 |
| *Asia* |  |  |  |  |
| Iran | 1 | – | 1 | 0.03 |
| Japan | 150 | 150 | 300 | 10.08 |
| Russia | 30 | 30 | 60 | 2.02 |
| *Australasia* |  |  |  |  |
| Australia | 108 | – | 108 | 3.63 |
| *Europe* |  |  |  |  |
| Croatia | 6 | 6 | 12 | 0.40 |
| Czech Republic | 5 | 40 | 45 | 1.51 |
| Denmark | 1 | – | 1 | 0.03 |
| Finland | – | 5 | 5 | 0.17 |
| Germany | 132 | 24 | 156 | 5.24 |
| Iceland | 17 | 17 | 34 | 1.14 |
| Ireland | – | 2 | 2 | 0.07 |
| Italy | 4 | 4 | 8 | 0.27 |
| Lithuania | – | 4 | 4 | 0.13 |
| Netherlands | 1 | – | 1 | 0.03 |
| Norway | 44 | 44 | 88 | 2.96 |
| Poland | 37 | 2 | 39 | 1.31 |
| Russia | 50 | 50 | 100 | 3.36 |
| Slovak Republic | 1 | – | 1 | 0.03 |
| Spain | 14 | – | 14 | 0.47 |
| Sweden | 36 | – | 36 | 1.21 |
| Switzerland | 12 | 12 | 24 | 0.81 |
| United Kingdom | 251 | 22 | 273 | 9.17 |
| *North America* |  |  |  |  |
| Canada | 110 | 127 | 237 | 7.96 |
| United States | 756 | 579 | 1335 | 44.86 |
| *Unclassified* |  |  |  |  |
| Russia | 42 | 42 | 84 | 2.82 |

Table S3. Model coefficients from basic and ecological models of the year slope (days yr^–1^). Geographic predictors included latitude and continent of the study site (in separate models), whilst ecological predictors included species’ migration distance category (short; long; unclassified), main breeding/passage habitat (forest; other), main diet type (invertebrate; other), habitat generalism (range: 1–9), diet generalism (range: 1–5) and the natural logarithm of body mass (g). Metric of spring migratory phenology (first arrival dates [FAD]; mean/median arrival dates [MED]), midyear decade in which the study was conducted, location of migrants’ arrival (breeding; passage; breeding or passage) and source of arrival data (citizen scientist observations; ornithological club reports; observatory observations; standardized capture and ringing at observatories; non-standardized field studies; standardized field studies) were also included as fixed effects in all models. Random effects included phylogeny, species, location (country of study), study, and species by location.

| Model | Fixed effect predictors | Posterior mean | 95% credibility interval | *P* MCMC |
| --- | --- | --- | --- | --- |
| Basic model (without geographic or ecological fixed effects) | Intercept* | –0.021 | –0.283, 0.249 | 0.872 |
|  | MED | –0.105 | –0.135, –0.072 | <0.001 |
|  | 1920 | –0.606 | –1.124, –0.093 | 0.019 |
|  | 1930 | –0.128 | –0.466, 0.220 | 0.457 |
|  | 1960 | –0.188 | –0.479, 0.103 | 0.199 |
|  | 1970 | –0.109 | –0.372, 0.147 | 0.393 |
|  | 1980 | –0.203 | –0.464, 0.051 | 0.129 |
|  | 1990 | –0.315 | –0.575, –0.049 | 0.024 |
|  | 2000 | –0.188 | –0.503, 0.112 | 0.223 |
|  | Arrival to passage ground | 0.025 | –0.009, 0.055 | 0.128 |
|  | Arrival to breeding or passage ground | 0.029 | –0.069, 0.120 | 0.549 |
|  | Citizen scientist observation | –0.019 | –0.172, 0.133 | 0.793 |
|  | Observatory observations | –0.049 | –0.242, 0.136 | 0.614 |
|  | Standardized capture/ringing at observatory | 0.077 | –0.045, 0.195 | 0.207 |
|  | Non-standardized field studies | 0.030 | –0.118, 0.170 | 0.676 |
|  | Standardized field studies | 0.071 | –0.037, 0.177 | 0.197 |
| Ecological model  (with latitude and ecological fixed effects) | Intercept* | 0.020 | –0.392, 0.442 | 0.927 |
|  | Northern latitude gradient | 0.001 | –0.004, 0.006 | 0.687 |
|  | Southern latitude gradient | 0.003 | –0.009, 0.015 | 0.609 |
|  | Southern hemisphere intercept | –0.073 | –0.635, 0.548 | 0.809 |
|  | Migration distance short | –0.030 | –0.056, –0.002 | 0.028 |
|  | Migration distance unclassified | 0.033 | –0.047, 0.117 | 0.424 |
|  | Diet generalism | 0.004 | –0.014, 0.020 | 0.638 |
|  | Habitat generalism | 0.000 | –0.009, 0.009 | 0.986 |
|  | Diet other | –0.001 | –0.034, 0.033 | 0.942 |
|  | Habitat forest | 0.000 | –0.029, 0.027 | 0.992 |
|  | Log (body mass) | –0.019 | –0.033, –0.005 | 0.010 |
|  | MED | –0.105 | –0.137, –0.073 | <0.001 |
|  | 1920 | –0.591 | –1.110, –0.069 | 0.027 |
|  | 1930 | –0.101 | –0.440, 0.262 | 0.585 |
|  | 1960 | –0.188 | –0.487, 0.118 | 0.222 |
|  | 1970 | –0.122 | –0.401, 0.134 | 0.355 |
|  | 1980 | –0.215 | –0.487, 0.048 | 0.120 |
|  | 1990 | –0.314 | –0.578, –0.034 | 0.030 |
|  | 2000 | –0.174 | –0.500, 0.134 | 0.269 |
|  | Arrival to passage ground | 0.029 | –0.002, 0.061 | 0.071 |
|  | Arrival to breeding or passage ground | 0.030 | –0.066, 0.130 | 0.549 |
|  | Citizen scientist observation | –0.025 | –0.180, 0.127 | 0.729 |
|  | Observatory observations | –0.039 | –0.236, 0.147 | 0.691 |
|  | Standardized capture/ringing at observatory | 0.080 | –0.054, 0.200 | 0.208 |
|  | Non-standardized field studies | –0.009 | –0.172, 0.147 | 0.913 |
|  | Standardized field studies | 0.073 | –0.048, 0.193 | 0.223 |
| Ecological model (with continent and ecological fixed effects) | Intercept* | 0.226 | –0.150, 0.599 | 0.231 |
|  | Continent Antarctica | 0.275 | –0.089, 0.635 | 0.118 |
|  | Continent Asia | 0.002 | –0.232, 0.243 | 0.978 |
|  | Continent Australasia | –0.137 | –0.408, 0.164 | 0.340 |
|  | Continent Europe | –0.117 | –0.338, 0.107 | 0.272 |
|  | Continent North America | –0.070 | –0.324, 0.183 | 0.588 |
|  | Migration distance short | –0.030 | –0.056, –0.002 | 0.033 |
|  | Migration distance unclassified | 0.033 | –0.043, 0.113 | 0.412 |
|  | Diet generalism | 0.004 | –0.014, 0.020 | 0.659 |
|  | Habitat generalism | 0.000 | –0.008, 0.009 | 0.974 |
|  | Diet other | 0.000 | –0.033, 0.034 | 0.991 |
|  | Habitat forest | 0.001 | –0.028, 0.030 | 0.959 |
|  | Log (body mass) | –0.019 | –0.033, –0.006 | 0.005 |
|  | MED | –0.105 | –0.137, –0.074 | <0.001 |
|  | 1920 | –0.566 | –1.069, –0.038 | 0.033 |
|  | 1930 | –0.068 | –0.397, 0.295 | 0.703 |
|  | 1960 | –0.231 | –0.524, 0.071 | 0.138 |
|  | 1970 | –0.160 | –0.423, 0.097 | 0.232 |
|  | 1980 | –0.250 | –0.506, 0.005 | 0.063 |
|  | 1990 | –0.332 | –0.585, –0.066 | 0.020 |
|  | 2000 | –0.204 | –0.508, 0.099 | 0.185 |
|  | Arrival to passage ground | 0.027 | –0.005, 0.060 | 0.097 |
|  | Arrival to breeding or passage ground | 0.002 | –0.099, 0.108 | 0.966 |
|  | Citizen scientist observation | –0.032 | –0.189, 0.112 | 0.665 |
|  | Observatory observations | –0.056 | –0.251, 0.139 | 0.566 |
|  | Standardized capture/ringing at observatory | 0.063 | –0.059, 0.185 | 0.305 |
|  | Non-standardized field studies | –0.084 | –0.257, 0.085 | 0.327 |
|  | Standardized field studies | 0.036 | –0.082, 0.153 | 0.543 |
| Model | Random effects | Variance | 95% credibility interval | |
| Basic model (without geographic or ecological fixed effects) | Phylogeny | 0.005 | 0.001, 0.010 | |
|  | Species | 0.002 | 0.001, 0.004 | |
|  | Location | 0.002 | 0.000, 0.009 | |
|  | Species by location | 0.002 | 0.000, 0.003 | |
|  | Study | 0.011 | 0.004, 0.021 | |
|  | Mean residual | 0.019 | 0.012, 0.027 | |
| Ecological model (with latitude and ecological fixed effects) | Phylogeny | 0.003 | 0.000, 0.007 | |
|  | Species | 0.003 | 0.001, 0.005 | |
|  | Location | 0.002 | 0.000, 0.011 | |
|  | Species by location | 0.002 | 0.000, 0.003 | |
|  | Study | 0.012 | 0.004, 0.023 | |
|  | Mean residual | 0.019 | 0.012, 0.028 | |
| Ecological model (with continent and ecological fixed effects) | Phylogeny | 0.002 | 0.000, 0.006 | |
|  | Species | 0.003 | 0.001, 0.005 | |
|  | Location | 0.002 | 0.000, 0.008 | |
|  | Species by location | 0.002 | 0.000, 0.004 | |
|  | Study | 0.011 | 0.004, 0.022 | |
|  | Mean residual | 0.019 | 0.011, 0.027 | |

* Intercept in basic models represent (i) FAD, (ii) decade 1910, (iii) arrival to breeding ground, and (iv) data from ornithological club reports. In ecological models the intercept additionally represent (v) 0° latitude OR unclassified continent, (vi) long-distance migrants, (vii) diet specialist, (viii) habitat specialist, (IX) invertebrate diet, (X) non-forest habitat, and (XI) 0 g mass.

Table S4. Model coefficients from basic and ecological models of temperature slope (days ºC^–1^). Geographic predictors included latitude and continent of the study site (in separate models), whilst ecological predictors included species’ migration distance category (short; long; unclassified), main breeding/passage habitat (forest; other), main diet type (invertebrate; other), habitat generalism (range: 1–9), diet generalism (range: 1–5) and the natural logarithm of body mass (g). Metric of spring migratory phenology (first arrival dates [FAD]; mean/median arrival dates [MED]) and source of arrival data (citizen scientist observations; ornithological club reports; observatory observations; standardized capture and ringing at observatories; non-standardized field studies; or standardized field studies) were also included as fixed effects in all models. Additionally, the location of temperature data (breeding; passage; breeding or passage; non-breeding) and location of migrants’ arrival (breeding; passage; breeding or passage) were included as a fixed effect in separate models. Random effects included phylogeny, species, location (country of study), study, and species by location.

| Model | Fixed effect predictors | Posterior | 95% credibility interval | P MCMC |
| --- | --- | --- | --- | --- |
| Basic model (without geographic or ecological fixed effects; with location of migrants’ arrival) | Intercept* | –1.384 | –2.051, –0.727 | <0.001 |
|  | MED | –0.472 | –0.791, –0.170 | 0.003 |
|  | Arrival to passage ground | 0.095 | –0.133, 0.318 | 0.409 |
|  | Arrival to breeding or passage ground | 0.257 | –0.280, 0.804 | 0.343 |
|  | Citizen scientist observation | 0.176 | –0.354, 0.756 | 0.472 |
|  | Observatory observations | 0.258 | –0.743, 1.321 | 0.586 |
|  | Standardized capture/ringing at observatory | 0.271 | –0.437, 0.933 | 0.428 |
|  | Non-standardized field studies | –0.022 | –0.817, 0.637 | 0.945 |
|  | Standardized field studies | 0.075 | –0.547, 0.697 | 0.813 |
| Basic model (without geographic or ecological fixed effects; with location of temperature data) | Intercept* | –1.367 | –2.087, –0.710 | <0.001 |
|  | MED | –0.475 | –0.802, –0.164 | 0.004 |
|  | Passage ground temperature | 0.095 | –0.136, 0.315 | 0.397 |
|  | Breeding or passage ground temperature | 0.260 | –0.283, 0.801 | 0.352 |
|  | Non-breeding ground temperature | 0.178 | –0.847, 1.353 | 0.724 |
|  | Citizen scientist observation | 0.143 | –0.466, 0.856 | 0.643 |
|  | Observatory observations | 0.231 | –0.902, 1.329 | 0.655 |
|  | Standardized capture/ringing at observatory | 0.245 | –0.502, 0.933 | 0.477 |
|  | Non-standardized field studies | –0.065 | –0.884, 0.670 | 0.856 |
|  | Standardized field studies | 0.053 | –0.655, 0.723 | 0.876 |
| Ecological model (with latitude and ecological fixed effects; with location of migrants’ arrival) | Intercept* | –1.647 | –3.828, 0.298 | 0.096 |
|  | Northern latitude gradient | 0.011 | –0.020, 0.045 | 0.475 |
|  | Migration distance short | –0.381 | –0.534, –0.239 | <0.001 |
|  | Migration distance unclassified | 0.083 | –0.340, 0.523 | 0.698 |
|  | Diet generalism | 0.009 | –0.079, 0.093 | 0.843 |
|  | Habitat generalism | 0.026 | –0.026, 0.080 | 0.333 |
|  | Diet other | –0.074 | –0.266, 0.096 | 0.406 |
|  | Habitat forest | –0.134 | –0.292, 0.017 | 0.080 |
|  | Log (body mass) | –0.048 | –0.114, 0.015 | 0.149 |
|  | MED | –0.466 | –0.807, –0.153 | 0.005 |
|  | Arrival to passage ground | 0.150 | –0.066, 0.381 | 0.178 |
|  | Arrival to breeding or passage ground | 0.352 | –0.261, 0.961 | 0.238 |
|  | Citizen scientist observation | 0.261 | –0.306, 0.881 | 0.337 |
|  | Observatory observations | 0.508 | –0.577, 1.680 | 0.331 |
|  | Standardized capture/ringing at observatory | 0.326 | –0.394, 1.055 | 0.372 |
|  | Non-standardized field studies | –0.221 | –1.080, 0.569 | 0.583 |
|  | Standardized field studies | 0.133 | –0.548, 0.808 | 0.687 |
| Ecological model (with latitude and ecological fixed effects; with location of temperature data) | Intercept* | –1.683 | –3.860, 0.268 | 0.086 |
|  | Northern latitude gradient | 0.011 | –0.019, 0.044 | 0.470 |
|  | Migration distance short | –0.381 | –0.531, –0.236 | <0.001 |
|  | Migration distance unclassified | 0.105 | –0.354, 0.577 | 0.653 |
|  | Diet generalism | 0.008 | –0.080, 0.097 | 0.859 |
|  | Habitat generalism | 0.026 | –0.025, 0.081 | 0.330 |
|  | Diet other | –0.074 | –0.254, 0.103 | 0.418 |
|  | Habitat forest | –0.133 | –0.291, 0.009 | 0.081 |
|  | Log (body mass) | –0.047 | –0.114, 0.018 | 0.156 |
|  | MED | –0.470 | –0.804, –0.133 | 0.006 |
|  | Passage ground temperature | 0.156 | –0.066, 0.384 | 0.172 |
|  | Breeding or passage ground temperature | 0.336 | –0.266, 0.987 | 0.269 |
|  | Non-breeding ground temperature | 0.501 | –0.661, 1.850 | 0.392 |
|  | Citizen scientist observation | 0.314 | –0.361, 1.039 | 0.349 |
|  | Observatory observations | 0.561 | –0.618, 1.935 | 0.339 |
|  | Standardized capture/ringing at observatory | 0.345 | –0.390, 1.156 | 0.355 |
|  | Non-standardized field studies | –0.219 | –1.095, 0.613 | 0.594 |
|  | Standardized field studies | 0.194 | –0.556, 0.925 | 0.607 |
| Ecological model (with continent and ecological fixed effects; with location of migrants’ arrival) | Intercept* | –0.808 | –2.093, 0.465 | 0.200 |
|  | Asia | 0.283 | –0.625, 1.142 | 0.496 |
|  | Europe | –0.202 | –1.114, 0.706 | 0.647 |
|  | North America | 0.451 | –0.624, 1.622 | 0.402 |
|  | Migration distance short | –0.373 | –0.519, –0.223 | <0.001 |
|  | Migration distance unclassified | –0.057 | –0.496, 0.401 | 0.794 |
|  | Diet generalism | 0.011 | –0.074, 0.100 | 0.807 |
|  | Habitat generalism | 0.028 | –0.027, 0.077 | 0.303 |
|  | Diet other | –0.074 | –0.266, 0.103 | 0.418 |
|  | Habitat forest | –0.134 | –0.284, 0.021 | 0.081 |
|  | Log (body mass) | –0.044 | –0.109, 0.019 | 0.185 |
|  | MED | –0.535 | –0.861, –0.212 | 0.002 |
|  | Arrival to passage ground | 0.122 | –0.100, 0.346 | 0.281 |
|  | Arrival to breeding or passage ground | 0.018 | –0.557, 0.620 | 0.948 |
|  | Citizen scientist observation | 0.139 | –0.443, 0.725 | 0.614 |
|  | Observatory observations | 0.039 | –1.082, 1.296 | 0.944 |
|  | Standardized capture/ringing at observatory | 0.150 | –0.512, 0.845 | 0.652 |
|  | Non-standardized field studies | –0.106 | –1.018, 0.750 | 0.813 |
|  | Standardized field studies | 0.165 | –0.564, 0.870 | 0.634 |
| Ecological model (with continent and ecological fixed effects; with location of temperature data) | Intercept* | –0.800 | –2.101, 0.477 | 0.224 |
|  | Asia | 0.293 | –0.650, 1.207 | 0.496 |
|  | Europe | –0.204 | –1.132, 0.749 | 0.659 |
|  | North America | 0.487 | –0.706, 1.687 | 0.397 |
|  | Migration distance short | –0.376 | –0.531, –0.233 | <0.001 |
|  | Migration distance unclassified | –0.077 | –0.541, 0.402 | 0.747 |
|  | Diet generalism | 0.010 | –0.081, 0.095 | 0.815 |
|  | Habitat generalism | 0.027 | –0.027, 0.078 | 0.315 |
|  | Diet other | –0.074 | –0.259, 0.108 | 0.426 |
|  | Habitat forest | –0.135 | –0.291, 0.018 | 0.082 |
|  | Log (body mass) | –0.045 | –0.109, 0.021 | 0.176 |
|  | MED | –0.544 | –0.869, –0.205 | 0.002 |
|  | Passage ground temperature | 0.128 | –0.094, 0.354 | 0.261 |
|  | Breeding or passage ground temperature | 0.018 | –0.596, 0.605 | 0.952 |
|  | Non-breeding ground temperature | –0.117 | –1.392, 1.193 | 0.842 |
|  | Citizen scientist observation | 0.104 | –0.545, 0.802 | 0.765 |
|  | Observatory observations | –0.013 | –1.299, 1.248 | 0.981 |
|  | Standardized capture/ringing at observatory | 0.133 | –0.565, 0.880 | 0.713 |
|  | Non-standardized field studies | –0.110 | –1.032, 0.810 | 0.797 |
|  | Standardized field studies | 0.149 | –0.587, 0.880 | 0.676 |
| Model | Random effects | Variance | 95% credibility interval | |
| Basic model (without geographic or ecological fixed effects) | Phylogeny | 0.076 | 0.005, 0.244 | |
|  | Species | 0.085 | 0.001, 0.148 | |
|  | Location | 0.173 | 0.000, 0.683 | |
|  | Species by location | 0.010 | 0.000, 0.053 | |
|  | Study | 0.112 | 0.009, 0.353 | |
|  | Mean residual | 0.128 | 0.040, 0.290 | |
| Basic model (without geographic or ecological fixed effects) | Phylogeny | 0.075 | 0.006, 0.256 | |
|  | Species | 0.084 | 0.000, 0.147 | |
|  | Location | 0.155 | 0.000, 0.683 | |
|  | Species by location | 0.010 | 0.000, 0.054 | |
|  | Study | 0.129 | 0.003, 0.405 | |
|  | Mean residual | 0.129 | 0.041, 0.290 | |
| Ecological model (with latitude and ecological fixed effects; with location of migrants’ arrival) | Phylogeny | 0.039 | 0.000, 0.136 | |
|  | Species | 0.069 | 0.000, 0.127 | |
|  | Location | 0.133 | 0.000, 0.761 | |
|  | Species by location | 0.014 | 0.000, 0.065 | |
|  | Study | 0.135 | 0.005, 0.400 | |
|  | Mean residual | 0.123 | 0.041, 0.275 | |
| Ecological model (with latitude and ecological fixed effects; with location of temperature data) | Phylogeny | 0.039 | 0.000, 0.135 | |
|  | Species | 0.070 | 0.001, 0.125 | |
|  | Location | 0.085 | 0.000, 0.703 | |
|  | Species by location | 0.014 | 0.000, 0.063 | |
|  | Study | 0.168 | 0.011, 0.456 | |
|  | Mean residual | 0.123 | 0.041, 0.275 | |
| Ecological model (with continent and ecological fixed effects; with location of migrants’ arrival) | Phylogeny | 0.041 | 0.000, 0.136 | |
|  | Species | 0.071 | 0.001, 0.128 | |
|  | Location | 0.014 | 0.000, 0.299 | |
|  | Species by location | 0.012 | 0.000, 0.059 | |
|  | Study | 0.160 | 0.026, 0.376 | |
|  | Mean residual | 0.124 | 0.041, 0.279 | |
| Ecological model (with continent and ecological fixed effects; with location of temperature data) | Phylogeny | 0.040 | 0.000, 0.140 | |
|  | Species | 0.072 | 0.000, 0.128 | |
|  | Location | 0.014 | 0.000, 0.284 | |
|  | Species by location | 0.012 | 0.000, 0.059 | |
|  | Study | 0.176 | 0.033, 0.413 | |
|  | Mean residual | 0.124 | 0.041, 0.276 | |

* Intercept in basic models represent (i) FAD, (ii) decade 1910, (iii) arrival to breeding ground OR breeding ground temperature, and (iv) data from ornithological club reports. In ecological models the intercept additionally represent (v) 0° latitude OR unclassified continent, (vi) long-distance migrants, (vii) diet specialist, (viii) habitat specialist, (IX) invertebrate diet, (X) non-forest habitat, and (XI) 0 g mass.

2. Bayesian models

Analyses were implemented in a Bayesian setting using the R package *MCMCglmm* (Hadfield 2010). R commands for priors in the basic and ecological models, respectively are:

prior.basic <– list(R=list(V=diag(6), nu=rep(0.002,6)), G=list(G1=list(V=1, nu=0.002),G2=list(V=1, nu=0.002),G3=list(V=1, nu=0.002),G4=list(V=1, nu=0.002),G5=list(V=1, nu=0.002)))

prior.ecological<– list(R=list(V=diag(6), nu=rep(0.002,6)), G=list(G1=list(V=1, nu=0.002),G2=list(V=1, nu=0.002),G3=list(V=1, nu=0.002),G4=list(V=1, nu=0.002),G5=list(V=1, nu=0.002)))

Using analyses of year slope as an example, the basic model was:

Year.basic<– MCMCglmm(Year_slope ~ Response_variable + Midyear_decade + Arrival_location + Data_source, random=~Animal + Species + Location + Study:Location + Study, data=Year.data, pedigree=tree, family=Gaussian, rcov=idh(Data_source):units, mev=SE^2, nitt=200000, burnin=150000, thin=500, prior=prior.basic)

The ecological model (including latitude and hemisphere interaction) was:

Year.ecological.lat<– MCMCglmm(Year_slope ~ Response_variable + Midyear_decade + Arrival_location + Data_source + Migration_distance + Main_diet + Main_habitat + Diet_generalism + Habitat_generalism + log(Body_size) + abs(Latitude)*Hemisphere, random=~Animal + Species + Location + Species:Location + Study, data=Year.data, pedigree=tree, family=Gaussian, rcov=idh(Data_source):units, mev=SE^2, nitt=200000, burnin=150000, thin=500, prior=prior.ecological)

The ecological model (including continent) was:

Year.ecological.cont<– MCMCglmm(Year_slope ~ Response_variable + Midyear_decade + Arrival_location + Data_source + Migration_distance + Main_diet + Main_habitat + Diet_generalism + Habitat_generalism + log(Body_size) + Continent, random=~Animal + Species + Location + Species:Location + Study, data=Year.data, pedigree=tree, family=Gaussian, rcov=idh(Data_source):units, mev=SE^2, nitt=200000, burnin=150000, thin=500, prior=prior.ecological)

In the above commands, ‘Year.data’ is the year slope data file read into R and ‘Year_slope’ is the slope estimate of migration timing over time. Response_variable, Midyear_decade, Arrival_location, Migration_distance, Main_diet, Main_habitat, Diet_generalism, Habitat_generalism and log(Body_size) are all fixed effect predictors. Random effects include ‘Animal’ defined by the phylogenetic relationship between the species in the dataset, ‘Species’ defined by the species scientific name according to BirdTree, ‘Location’ defined by the country of study site, ‘Species:Location’ which defined each species by location combination, and ‘Study’ defined by the reference name.

Reference:

Hadfield, J.D. (2010) MCMC methods for multi–response generalized linear mixed models: the MCMCglmm R package. *Journal of Statistical Software*, **33**, 1–22.
